# Effects of Acetaminophen Exposure on Outcomes of Patients Receiving Immune Checkpoint Inhibitors for Advanced Non-Small-Cell Lung Cancer: A Propensity Score-Matched Analysis

**DOI:** 10.3390/curroncol30090589

**Published:** 2023-09-01

**Authors:** Fabrizio Nelli, Antonella Virtuoso, Diana Giannarelli, Agnese Fabbri, Julio Rodrigo Giron Berrios, Eleonora Marrucci, Cristina Fiore, Enzo Maria Ruggeri

**Affiliations:** 1 Thoracic Oncology Unit, Department of Oncology and Hematology, Central Hospital of Belcolle, 01100 Viterbo, Italy; 2 Biostatistics Unit, Scientific Directorate, Fondazione Policlinico Universitario A. Gemelli, Istituto di Ricovero e Cura a Carattere Scientifico (IRCCS), 00168 Rome, Italy; 3 Medical Oncology Unit, Department of Oncology and Hematology, Central Hospital of Belcolle, 01100 Viterbo, Italy

**Keywords:** non-small-cell lung cancer, immune checkpoint inhibitors, first-line therapy, second-line therapy, acetaminophen, disease control benefit, survival

## Abstract

(1) Background: Several studies have investigated potential interactions between immune checkpoint inhibitors (ICIs) and commonly prescribed medications. Although acetaminophen (APAP) has not been considered susceptible to interaction with ICIs, recent research has shown that detectable plasma levels of this drug can hinder the efficacy of PD-1/PD-L1 blockade therapies. A reliable assessment of the potential interaction between APAP and ICIs in advanced non-small cell lung cancer (NSCLC) patients would be worthwhile since it is often prescribed in this condition. We sought to evaluate the impact of the concomitant use of APAP in patients with advanced NSCLC on PD-1/PD-L1 blockade using real-world evidence. (2) Methods: This study included consecutive patients with histologically proven stage IV NSCLC who underwent first-line therapy with pembrolizumab as a single agent or in combination with platinum-based chemotherapy, or second-line therapy with pembrolizumab, nivolumab, or atezolizumab. The intensity of APAP exposure was classified as low (therapeutic intake lasting less than 24 h or a cumulative intake lower than 60 doses of 1000 mg) or high (therapeutic intake lasting more than 24 h or a total intake exceeding 60 doses of 1000 mg). The favorable outcome of anti-PD-1/PD-L1 therapies was defined by durable clinical benefit (DCB). Progression-free survival (PFS) and overall survival (OS) were relevant to our efficacy analysis. Propensity score matching (PSM) methods were applied to adjust for differences between the APAP exposure subgroups. (3) Results: Over the course of April 2018 to October 2022, 80 patients were treated with first-line pembrolizumab either as single-agent therapy or in combination with platinum-based chemotherapy. During the period from June 2015 to November 2022, 145 patients were given anti-PD-1/PD-L1 blockade therapy as second-line treatment. Subsequent efficacy analyses relied on adjusted PSM populations in both treatment settings. Multivariate testing revealed that only the level of APAP and corticosteroid intake had an independent effect on DCB in both treatment lines. Multivariate Cox regression analysis confirmed high exposure to APAP and immunosuppressive corticosteroid therapy as independent predictors of shorter PFS and OS in both treatment settings. (4) Conclusions: Our findings would strengthen the available evidence that concomitant intake of APAP blunts the efficacy of ICIs in patients with advanced NSCLC. The detrimental effects appear to depend on the cumulative dose and duration of exposure to APAP. The inherent shortcomings of the current research warrant confirmation in larger independent series.

## 1. Introduction

Over the past decade, advances in molecular characterization and the introduction of new drugs have radically reshaped the therapeutic landscape of advanced non-small cell lung cancer (NSCLC) [1]. Immune checkpoint inhibitors (ICIs) are currently the gold standard of care for patients whose malignancies lack actionable driver mutations in first- and second-line therapy [2]. Although ICIs targeting the programmed cell death-1 (PD-1) and PD-ligand (L)-1 pathways improved outcomes compared to cytotoxic chemotherapy, a long-term survival benefit is limited to less than 30% of patients with advanced NSCLC [3,4]. This discrepancy remains an unmet need and drives clinical research to identify predictive biomarkers that can improve the therapeutic index of ICIs [5]. Since lung cancer patients are diagnosed with pre-existing comorbidities, the prescription of multiple baseline medications is a frequent occurrence [6]. Several researchers have investigated potential interactions between ICIs and commonly prescribed medications in light of the evidence that they can engage the immune system through a variety of mechanisms [7,8,9]. In this regard, concomitant therapies with corticosteroids [10,11], antibiotics [12,13], proton pump inhibitors (PPIs) [14,15,16], oral opioids [14], and metformin [15] have been shown to impair the efficacy of agents targeting the PD-1/PD-L1 checkpointin advanced NSCLC. Conversely, prescriptions of statins [16], fibrates [17], beta-blockers [18], nonsteroidal anti-inflammatory drugs (NSAIDs) [19], and angiotensin-converting enzyme inhibitors (ACEi) or angiotensin II type 2 receptor blockers (ARBs) [20] have been associated with improved outcomes. Given that most of these studies relied on real-world retrospective series, the evidence provided is weak. Even meta-analytic efforts to improve data consistency have not resulted in avoiding or recommending a specific concomitant medication in patients on PD-1/PD-L1 blockade [21,22,23,24].

Despite none of the prior investigations considered acetaminophen (N-acetyl-p-aminophenol, APAP) susceptible to interaction with ICIs, recent research has shown that detectable plasma levels of this drug at the onset of PD-1 inhibition were associated with worse survival outcomes [25]. This study has provided consistent evidence for the role of APAP as a suppressor of antitumor immunity, but this hypothesis requires further investigation. A reliable assessment of the potential interaction between ICI-based therapies and APAP in advanced NSCLC patients would be worthwhile since this medication is often prescribed for its antipyretic and analgesic properties [26]. We, therefore, sought to evaluate the impact of the concomitant use of APAP in patients with advanced NSCLC receiving PD-1/PD-L1-targeted agents, using real-world evidence from medical records available from our institutional database and registry of government agencies for monitoring high-cost drug prescriptions.

## 2. Materials and Methods

### 2.1. Study Design and Participants

We conducted a retrospective investigation in a real-world, single-center setting to assess the influence of APAP intake on ICI outcomes by weighing its relationship with baseline clinical characteristics and other concurrent medications. This study included consecutive patients with histologically proven stage IV NSCLC who underwent first-line therapy with pembrolizumab as a single agent or in combination with platinum-based chemotherapy or second-line therapy with pembrolizumab, nivolumab, or atezolizumab. All anti-PD-1/PD-L1 agents were administered according to standard indications and were prescribed at doses and schedules specified by their respective labels. Patients with EGFR, ALK, or ROS1 activating mutations were excluded. Patients with symptomatic brain metastases were eligible after radiotherapy if they had neurologically returned to baseline 14 days before the treatment began.

To mitigate the impact of immortal time bias, concomitant APAP intake was defined as that of patients who had an active prescription in the window between 30 days before and 90 days after the first ICI infusion. The intensity of APAP exposure was classified as low (LAE, therapeutic intake lasting less than 24 h or a cumulative intake lower than 60 doses of 1000 mg) or high (HAE, therapeutic intake lasting more than 24 h or a total intake exceeding 60 doses of 1000 mg). Based on their presumed effect on ICI outcomes in cancer patients, we also addressed the following intake categories of concomitant drugs: corticosteroids (dose ≥10 mg prednisone equivalent per day for at least 5 days dosing in the window between 30 days before and 90 days after ICI treatment initiation, excluding premedications to chemotherapy; yes vs. no), systemic antibiotics in the window between 30 days before and 90 days after ICI treatment initiation (yes vs. no), baseline PPIs (yes vs. no), baseline statins (yes vs. no), baseline fibrates (yes vs. no), baseline NSAIDs and/or acetylsalicylic acid (yes vs. no), baseline beta-blockers (yes vs. no), baseline ACEi/ARBs (yes vs. no), baseline metformin (yes vs. no), and baseline oral or transdermal opioids (yes vs. no).

The clinical outcomes relevant tothe purposes of this study included objective response rate, progression-free survival (PFS), and overall survival (OS). Patients underwent a baseline evaluation of disease extent within four weeks of beginning treatment. Subsequent evaluations were scheduled every 12 to 16 weeks, according to the monitoring requirements of the national drug regulatory agency [27]. We reassessed all radiology records of included patients to determine changes in the extent of disease using the Response Evaluation Criteria in Solid Tumors (RECIST 1.1) [28]. The favorable outcome of PD-1/PD-L1-targeted therapies relied on evidence of durable clinical benefit (DCB), which indicated any objective response or stable disease lasting more than six months. This group (DCB, durable clinical benefit) was therefore compared to those who did not experience a durable clinical benefit (NCB group, progressive disease, or stable disease lasting less than six months). PFS refers to the elapsed time from the start of treatment with an anti-PD-1/PD-L1 agent to its permanent discontinuation for any reason). OS referred to the elapsed time from the start of treatment with an anti-PD-1/PD-L1 agent to death for any reason). Patients who did not progress or die as of the last follow-up time were censored (cut-off date 6 May 2023).

The procedures adopted in this research complied with the Strengthening the Reporting of Observational Studies in Epidemiology (STROBE) guidelines. The relevant Ethics Committee granted formal approval for this study (Oss-R-281; approval code: 855/CE Lazio1). The data on which this investigation is based were obtained for clinical purposes and gathered from electronic medical and pharmacy charts. All participants provided written informed consent before receiving any active treatment and for the processing of unidentified clinical information for subsequent research purposes.

### 2.2. Statistical Analysis

A mean with standard deviation (SD) was used to describe normally distributed variables, while a median with a 95% confidence interval (CI) or interquartile range (IQR) was reported for skewed variables. Patient characteristics were classified according to treatment setting (first vs. second-line therapy) and intensity of APAP exposure (LAE vs. HAE). Differences at baseline between the APAP exposure subgroups in the crude population were compared using Pearson’s *χ*^2^ test for categorical data and Mann–Whitney’s *U* test for continuous variables. Propensity score matching (PSM) methods were applied to adjust for differences between the APAP exposure subgroups. Propensity scores were calculated using a logistic regression model, including variables that showed a significant imbalance in comparisons at baseline. A propensity score–matched cohort was created in both first- and second-line treatment settings. The nearest neighbor method was applied using 1:1 matching, without replacement, and with a caliper width equal to 0.1. After calculating standardized differences between adjusted covariates, differences less than or equal to 0.1 were considered acceptably balanced. A Fisher’s exact test allowed for univariate comparisons to examine the correlation between clinical variables and DCB. The significant variables from the univariate analysis were then considered to estimate the odds ratio (OR) of DCB with a 95% confidence interval (CI) through a multivariate logistic regression model. The Kaplan–Meier curves generated for PFS and OS of different patient subgroups were compared using the log-rank test. Hazard ratios (HRs) with 95% CI for PFS and OS were analyzed using multivariable Cox regression models that were adjusted for age, sex, Eastern Cooperative Oncology Group Performance Status (ECOG PS), histology, body mass index (BMI), and disease burden categories. All findings were considered statistically significant with a two-sided test at a *p*-value <0.05. Statistical analyses were conducted using SPSS (IBM SPSS Statistics for Windows, version 23.0, Armonk, NY, USA). Figure rendering was performed with Prism software (GraphPad, version 9). R software version 4.1.2 (The R Foundation for Statistical Computing, Vienna, Austria) nd the MatchIt library extension allowed for PSM [29].

## 3. Results

### 3.1. Patient Characteristics

The current analysis included 225 consecutive cases. Over the course of April 2018 to October 2022, 80 patients were treated with first-line pembrolizumab either as single-agent therapy (if their PD-L1 TPS was ≥50%) or in combination with platinum-based chemotherapy (if their PD-L1 TPS was <50%). During the period from June 2015 to November 2022, 145 patients were given anti-PD-1/PD-L1 blockade therapy (including nivolumab, pembrolizumab, or atezolizumab) as second-line treatment after they had received upfront platinum-based chemotherapy. In both settings, all patients had metastatic disease extension and had undergone at least two cycles of treatment. Clinical and pathological features were mostly homogeneous across the treatment cohorts based on APAP exposure level. However, we observed unbalanced distributions of ECOG PS2 and corticosteroid use in the first-line setting, as well as corticosteroid and opioid prescriptions in the second-line setting. Because these variables are presumed to have a detrimental effect on outcomes, subsequent efficacy analyses relied on adjusted PSM populations in both treatment settings. Table 1 and Table 2 depict the baseline characteristics of patients receiving first- and second-line treatment, respectively.

### 3.2. Clinical Benefit Analysis

The median duration of treatment for patients who received first-line therapy was sevencycles (range 2–35). In this population, we observed 15 (28.8%) partial responses, 17 (32.7%) disease stabilizations, 13 (25.0%) of which lasted more than six months, and 20 (38.4%) progressions of disease. As a result, DCB and NCB were reported in 27 (51.9%) and 25 (48.1%) cases, respectively. The univariate analysis found that prescriptions of corticosteroids at immunosuppressive dosages and high exposure to APAP correlated significantly with treatment failure. On multivariate analysis, both pharmacological variables retained their predictive significance. Patients who were treated with PD-1/PD-L1 targeted agents as second-line therapy had a median treatment length of 6 cycles (range 2-91). Among these patients, 26 (24.1%) achieved a partial response, 32 (29.6%) experienced disease stabilization, 20 (18.5%) of which lasted longer than six months, and 50 (46.3%) resulted in progression. Thus, DCB and NCB were found in 46 (42.6%) and 62 (57.4%) of cases, respectively. The results of a univariate comparative assessment revealed that having a disease burden with more than two metastatic sites, bone metastases, immunosuppressive corticosteroid dosing, and high exposure to APAP were significantly linked to not achieving DCB. The multivariate testing confirmed that only pharmacological covariates had an independent effect on DCB, which is consistent with the findings arising from the context of first-line therapy. Table 3 details the analysis of DCB in both first- and second-line treatment settings.

### 3.3. Survival Analysis

**Figure 1:**
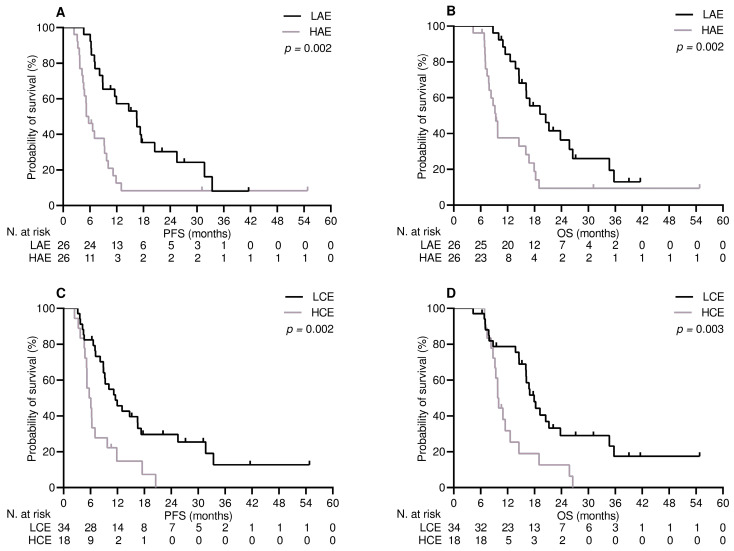
First-line treatment survival outcomes depend on significant variables. (**A**) Progression-free survival: low-level acetaminophen exposure (LAE) vs. high-level acetaminophen exposure (HAE); (**B**) overall survival: low-level acetaminophen exposure (LAE) vs. high-level acetaminophen exposure (LAE); (**C**) progression-free survival: low-level corticosteroid exposure (LCE) vs. high-level corticosteroid exposure (HCE); and (**D**) overall survival: low-level corticosteroid exposure (LCE) vs. high-level corticosteroid exposure (HCE).

**Figure 2:**
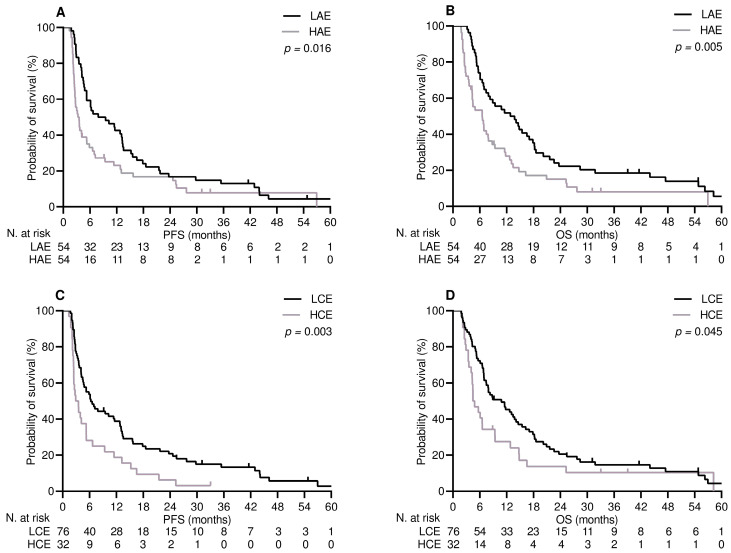
Second-line treatment survival outcomes depend on significant variables. (**A**) progression-free survival: low-level acetaminophen exposure (LAE) vs. high-level acetaminophen exposure (HAE); (**B**) overall survival: low-level acetaminophen exposure (LAE) vs. high-level acetaminophen exposure (LAE); (**C**) progression-free survival: low-level corticosteroid exposure (LCE) vs. high-level corticosteroid exposure (HCE); (**D**) overall survival: low-level corticosteroid exposure (LCE) vs. high-level corticosteroid exposure (HCE).

After a median follow-up time of 17.8 months (95% CI 10.8–24.7), out of the 52 patients receiving the first line of therapy, 9 (17.3%) were still being treated, 43 (82.7%) discontinued their treatment due to disease progression, 40 (76.9%) died, and 12 (23.1%) were censored at the cut-off date. The median PFS and OS in this population were 9.2 months (95% CI 6.9–11.4) and 14.7 months (95% CI 8.8–20.7), respectively. The median duration of follow-up in patients who received the second line of therapy was 34.1 months (95% CI 28.8–39.4). Eight (7.4%) remained on treatment, 100 (92.5%) withdrew as a result of progressive disease, 97 (89.8%) died, and 11 (10.1%) were censored at the study cut-off. The median PFS and OS in these patients were 5.2 months (95% CI 4.1–6.6) and 7.9 months (95% CI 6.5–11.2), respectively. Achieving DCB resulted in a significant improvement in terms of PFS (Supplementary Figure S1) and OS (Supplementary Figure S2) in both treatment settings. Based on these findings, variables related to DCB are likely to affect PFS and OS [30]. In patients who received treatment in the first-line setting, multivariate analysis revealed high exposure to APAP and immunosuppressive corticosteroid therapy as independent predictors of shorter PFS and OS (Table 4 and Figure 1). The same testing for patients undergoing second-line therapy confirmed that the level of APAP exposure and receipt of corticosteroids at immunosuppressive dosages had an independent impact on both survival outcomes (Table 5 and Figure 2).

## 4. Discussion

We described the results of a retrospective analysis of the impact of concomitant APAP intake in terms of clinical benefit and survival outcomes in patients with advanced NSCLC on PD-1/PD-L1 blockade. Accordingly, a high level of APAP exposure in the window between 30 days before and 90 days after the first cycle of ICI therapy resulted in an increased risk of treatment failure and a shorter duration of PFS and OS. Multivariate analyses confirmed the detrimental effect of a pronounced intake of APAP on disease outcomes in both first- and second-line settings. Similar findings have not been reported previously in this specific patient population and require a critical appraisal of their clinical relevance.

The current research relies on a retrospective single-center analysis of real-life data, implying inherent strengths and weaknesses. Real-world studies have become a meaningful tool in cancer research, as they provide different stakeholders with evidence that can bridge the gap between clinical trials and routine practice [31]. In this regard, the retrospective analysis methodology of this study sought to address the issue of the predictive potential of APAP exposure, which would be unlikely to be the subject of prospective research [32]. The collection of data at a single center allowed for a comprehensive review of medical and pharmaceutical prescriptions of several concomitant drugs over a wide time frame. The reliability of the clinical records in our series is ensured by their close matching with the government registry for monitoring the reimbursement of high-cost drugs (including pembrolizumab, nivolumab, and atezolizumab) [27]. In addition, we conducted a PSM of our series in accordance with best practice guidelines for medical research, distinguishing first- and second-line treatments for their intrinsic prognostic value and taking into account all potentially clinical–pathological and pharmacological predictive covariates [33,34]. The present investigation expands on the results of the study by Bassede et al., which was the first and only in humans to show evidence of APAP as a suppressor of the anticancer immune response [25]. The viability of our conclusions must rely on comparison, albeit indirect, with the clinical and methodological aspects of this pivotal study. The French authors provided post hoc analyses of data from three prospective trials that enrolled patients with different tumor types and were treated with anti-PD-1/PD-L1 agents or their combination with anti-CTLA-4 monoclonal antibodies. Detectable serum APAP or its metabolites were found to be associated with poorer response rates and survival outcomes regardless of dosing. A key difference in our study concerns the inclusion criteria. We enrolled consecutive patients with a uniform diagnosis and selected homogeneously by therapeutic indication. Exposure to APAP in our series was based on the level of intake according to relevant toxicological standards [35,36]. Low to moderate dosing in the time window under study did not appear to affect disease outcomes, which were consistent with those expected from real-world evidence. By contrast, a high level of APAP intake independently predicted a worse response and shorter survival duration. This striking difference suggests a dose-dependent interaction that may relate to the immunomodulatory effects of APAP. Bassede et al. reported strong and convincing evidence that exposure to APAP dampens the efficacy of ICIs through a counterproductive effect on host immune responses. The researchers demonstrated, in mouse models and in healthy volunteers and cancer patients, that APAP is able to induce up-regulation of regulatory T cells (Treg) and signaling of their soluble stimulatory mediator, interleukin (IL)-10. Despite being consistent with earlier ex vivo evidence, these findings have not been confirmed in the clinical setting [37,38,39]. In general, human studies provide conflicting results concerning the immunomodulatory effects of APAP in infectious diseases [40]. Relevant studies have shown that administration of APAP immediately before or at the time of vaccination may impair immunization reactogenicity and humoral immunogenicity [41,42]. As a result, the World Health Organization [43] and the Center for Disease Control [44] advise against administering APAP as a preventative measure to treat febrile reactions associated with vaccination. Recently, there has been an urgent need to determine whether the use of over-the-counter drugs such as APAP has a negative impact on the immunogenicity and efficacy of severe acute respiratory syndrome coronavirus 2 (SARS-CoV-2) vaccines [45]. Although some changes in antibody responses may result from the intake of these drugs, there is no evidence that their prescription to manage side effects has any relevant impact on T-cell responses and subsequent vaccine efficacy [46]. While the clinical implications of interactions between APAP and systemic immunity remain unclear, the dual role of the immune response in APAP-induced liver injury can provide additional insights [47]. Following excessive exposure to APAP, liver tissues are enriched with different T lymphocytes, including CD4^+^T helper and Treg cells, CD8^+^T cytotoxic cells, and γδ T cells [48]. In this condition, T helper 1 cells promote the release of IL-2 and interferon-γ and stimulate Treg cells to produce anti-inflammatory mediators such as IL-10 and TGF-β, which conversely attenuate immune-mediated liver damage and inhibit the systemic immune response [37,49]. The disruption of T cell homeostasis and implications for systemic immunity induced by incremental APAP dosing seems consistent with the results obtained by Bassede et al. [25] and may account for the dose-dependent interaction described in the present research. The most important issue in our analysis remains the potential for confounding. Although the reasons for the use of APAP do not differ across intake cohorts, we cannot rule out that the need for higher dosing indicates a more aggressive cancer disease with a less favorable prognosis. In an attempt to mitigate this effect, we conducted comprehensive PSM and multivariate analyses. Evidence that higher dosing of APAP and the need for intake of corticosteroids at immunosuppressive dosages have a similar independent impact on the survival of upfront-treated patients with a better prognosis would suggest an interaction at the immunologic level with ICI-based treatments. However, since the intensity of cancer-related pain is a well-known prognostic factor, it is difficult to tease out that patients requiring a higher dosage of APAP are expected to have a worse outcome [50]. Given the inherent imbalance of these variables and the lack of comparable data, our results seem to further enrich the controversy over whether concomitant medications have a causal or purely associative effect on adverse prognostic features [51].

The current study acknowledges additional shortcomings, including but not limited to the following issues. Although the retrospective, monocentric design made it easier to have a thorough examination of medical and pharmacy records, it does so by mirroring an internal prescribing procedure that might not be applicable elsewhere. To avoid selection bias, we enrolled patients consecutively, excluding those who had received less than two cycles of treatment. This subgroup represents a considerable proportion of patients with dismal prognoses, and their exclusion may have favorably influenced the evaluation of some correlations [52]. Furthermore, we were unable to ensure an independent reassessment of the radiology records, resulting in a potential overestimation of objective response rates. The same imaging of the included patients was not reinterpreted according to the immune-related criteria of treatment response [53]. Because of these constraints, the activity of anti-PD-1/PD-L1 therapies and the duration of PFS may have been inaccurately estimated. Finally, the most important methodological flaws of this study are the sample size and the duration of follow-up after first-line treatment, both of which are limited. PSM mitigated potential confounders from selection bias, but resulted in a numerical reduction of cases in both treatment settings. The power of the study in detecting statistically significant differences is consequently blunted. This also implies that multivariate statistical analyses may increase the occurrence of false-positive results. Their significance should be properly considered as a means of generating research hypotheses.

## 5. Conclusions

APAP, as a single agent or in combination with opioids or NSAIDs, is the most common therapeutic resource for the treatment of cancer-related pain [54]. Relevant guidelines recommend its use as a preferred option for the relief of mild to moderate symptoms [55] and for the prevention or treatment of some ICI-related reactions [56]. Unprecedented results of comprehensive research have shed light on a possible deleterious effect of APAP intake on the effectiveness of ICIs [25]. Although our results confirm this clinical concern, they suggest that only a pronounced and/or prolonged intake of APAP would be able to hamper the immune response to anti-PD-1/PD-L1 agents in patients with advanced NSCLC. A more restrained and discontinuous intake (less than four doses of 1000 mg per week) at the start of treatment and over the following three months does not seem to have a worsening effect on expected outcomes [57]. The inherent limitations of the present research and the lack of additional studies for proper comparison imply that these results should be considered exploratory. Whether these conclusions are applicable to other cancer types or different therapeutic settings requires further investigation.

## Figures and Tables

**Table 1:** Patient characteristics in a first-line treatment setting.

Variable	All Patients, N = 80 (100%)	General Population			PSM Population		
		LAE, N = 45 (100%)	HAE, N = 35 (100%)	*p* Value	LAE, N = 26 (100%)	HAE, N = 26 (100%)	*p* Value
Age							
Mean (SD), years	67.0 (8.8)	68.0 (8.3)	65.7 (9.4)	0.299	70.1 (9.2)	66.7 (8.7)	0.128
≥70 years	32 (40.0%)	20 (44.4%)	12 (34.3%)	0.358	15 (57.7%)	10 (38.5%)	0.165
Sex				0.923			0.749
female	21 (26.2%)	12 (26.7%)	9 (20.0%)		6 (23.1%)	7 (26.9%)	
male	59 (73.8%)	33 (73.3%)	26 (80.0%)		20 (76.9%)	19 (73.1%)	
ECOG PS				0.018			0.999
0 or 1	68 (85.0%)	42 (93.3%)	26 (74.3%)		23 (88.5%)	23 (88.5%)	
2	19 (15.0%)	3 (6.7%)	9 (25.7%)		3 (11.5%)	3 (11.5%)	
Histology				0.670			0.442
Squamous	10 (12.5%)	5 (11.1%)	5 (14.3%)		3 (11.5%)	5 (19.2%)	
Nonsquamous	70 (87.5%)	40 (88.9%)	30 (85.7%)		23 (88.5%)	21 (80.8%)	
No. of metastatic sites				0.413			0.158
≤2	43 (53.7%)	26 (57.8%)	19 (54.3%)		18 (69.2%)	13 (50.0%)	
>2	37 (46.3%)	17 (42.3%)	18 (45.7%)		8 (30.8%)	13 (50.0%)	
Bone metastases				0.757			0.999
Not present	63 (78.8%)	36 (80.0%)	27 (77.2%)		22 (84.6%)	22 (84.6%)	
Any	17 (21.2%)	9 (20.0%)	8 (22.8%)		4 (15.4%)	4 (15.4%)	
CNS metastases				0.923			0.337
Not present	59 73.8%)	33 (73.4%)	26 (74.3%)		21 (80.8%)	18 (69.2%)	
Any	21 (26.2%)	12 (26.6%)	9 (25.7%)		5 (19.2%)	8 (30.8%)	
Liver metastases				0.060			0.124
Not present	72 (90.0%)	43 (95.6%)	29 (82.9%)		24 (92.3%)	20 (76.9%)	
Any	8 (10.0%)	2 (4.4%)	6 (17.1%)		2 (7.7%)	6 (23.1%)	
PD-L1 TPS				0.363			0.798
<1%	32 (40.0%)	21 (46.7%)	11 (31.4%)		5 (19.2)	7 (26.9%)	
≥1% and ≤49%	11 (13.7%)	6 (13.3%)	5 (14.3%)		3 (11.5%)	3 (11.5%)	
≥50%	37 (46.2%)	18 (40.0%)	19 (54.3%)		18 (69.2%)	16 (61.5%)	
BMI							
Mean (SD), (kg/m^2^)	25.6 (5.2)	25.9 (5.1)	25.2 (5.4)	0.485	26.4 (5.5)	25.4 (5.5)	0.577
≥25	41 (51.2%)	26 (57.8%)	15 (42.8)	0.185	14 (53.8%)	11 (42.3%)	0.405
Smoking habit				0.141			0.638
Never	9 (11.2%)	3 (6.7%)	6 (17.1%)		2 (7.7%)	3 (11.5%)	
Ever	71 (88.8%)	42 (93.3%)	29 (82.9%)		24 (93.3%)	23 (88.5%)	
Type of treatment				0.265			0.351
Pembrolizumab	42 (52.5%)	21 (46.7%)	21 (60.0%)		18 (69.2%)	17 (65.4%)	
Pemetrexed-based	33 (41.2%)	22 (48.9%)	11 (31.4%)		8 (30.8%)	7 (26.9%)	
Paclitaxel-based	5 (6.3%)	2 (4.4)	3 (8.6%)		-	2 (7.7%)	
Reasons for APAP intake				0.377			0.704
Cancer-related	37 (46.2%)	18 (40.0%)	19 (54.3%)		11 (42.3%)	14 (53.8%)	
Treatment-related	24 (30.0%)	16 (35.6%)	8 (22.8%)		9 (34.6%)	7 (26.9%)	
Others	19 (23.8%)	11 (24.4%)	8 (22.8%)		6 (23.1%)	5 (19.2%)	
Corticosteroids (yes)	24 (30.0%)	8 (17.8%)	16 (45.7%)	0.007	8 (30.8%)	10 (38.5%)	0.560
Systemic antibiotics (yes)	13 (16.2%)	9 (20.0%)	4 (11.4%)	0.303	6 (23.1%)	3 (11.5%)	0.271
PPI (yes)	39 (48.7%)	22 (48.9%	17 (48.5%)	0.978	15 (57.7%)	9 (34.6%)	0.095
Statins (yes)	20 25.0%)	11 (24.4%)	9 (25.7%)	0.896	7 (26.9%)	7 (26.9%)	0.999
Fibrates (yes)	8 (10.0%)	3 (6.7%)	5 (14.3%)	0.260	2 (7.7%)	4 (15.4%)	0.385
NSAIDs or ASA (yes)	28 (35.0%)	15 (33.3%)	13 (28.9%)	0.723	9 (34.6%)	11 (42.3%)	0.569
Beta-blockers (yes)	24 (30.0%)	13 (28.9%)	11 (31.4%)	0.806	8 (30.8%)	10 (38.5%)	0.560
ACEi or ARBs (yes)	35 (43.7%)	16 (35.6%)	19 (54.3%)	0.094	9 (34.6%)	12 (46.2%)	0.397
Metformin (yes)	10 (12.5%)	5 (11.1%)	5 (14.3%)	0.670	2 (7.7%)	4 (15.4%)	0.385
Oral or transdermal opioids (yes)	32 (40.0%)	16 (35.6%)	16 (45.7%)	0.358	10 (38.5%)	12 (46.2%)	0.575

LAE, low-level acetaminophen (APAP) exposure cohort; HAE, high-level APAP exposure cohort; PSM, propensity score matching; SD, standard deviation; ECOG PS, Eastern Cooperative Oncology Group Performance Status; CNS, central nervous system; PD-L1 TPS, programmed cell death ligand-1 tumor proportion score; BMI, body mass index; PPI, proton pump inhibitors; NSAIDs, nonsteroidal anti-inflammatory drugs; ASA, acetylsalicylic acid; ACEi, angiotensin-converting enzyme inhibitors; and ARBs, angiotensin II type 2 receptor blockers.

**Table 2:** Patient characteristics in a second-line treatment setting.

Variable	All Patients, N = 145 (100%)	General Population			PSM Population		
		LAE, N = 70 (100%)	HAE, N = 75 (100%)	*p* Value	LAE, N = 54 (100%)	HAE, N = 54 (100%)	*p* Value
Age							
Mean (SD), years	70.1 (8.9)	69.2 (9.9)	70.9 (8.0)	0.375	67.5 (10.9)	70.3 (8.2)	0.209
≥70 years	89 (61.4%)	43 (61.4%)	46 (61.3%)	0.991	29 (53.7%)	31 (57.4%)	0.698
Sex				0.058			0.380
Female	44 (30.3%)	16 (22.9%)	28 (37.3%)		12 (22.2%)	16 (29.6%)	
Male	101 (69.7%)	54 (77.1%)	47 (69.7%)		42 (77.8%)	38 (70.4%)	
ECOG PS				0.497			0.421
0 or 1	116 (80.6%)	58 (82.9%)	58 (78.4%)		46 (85.2%)	42 (77.8%)	
2	28 (19.4%)	12 (17.1%)	16 (21.6%)		8 (14.8%)	12 (22.2%)	
Histology				0.549			0.683
Squamous	47 (32.4%)	21 (30.0%)	26 (34.7%)		17 (31.5%)	19 (35.2%)	
Nonsquamous	98 (67.6%)	49 (70.0%)	49 (65.3%)		37 (68.5%)	35 (64.8%)	
No. of metastatic sites				0.325			0.245
≤2	83 (57.2%)	43 (61.4%)	40 (53.3%)		33 (61.1%)	27 (50.0%)	
>2	62 (42.8%)	27 (38.6%)	35 (46.7%)		21 (38.9%)	27 (50.0%)	
Bone metastases				0.562			0.814
Not present	113 (79.1%)	56 (80.0%)	57 (76.0%)		43 (79.6%)	42 (77.8%)	
Any	32 (22.1%)	14 (20.0%)	18 (24.0%)		11 (20.4%)	12 (22.2%)	
SNC metastases				0.659			0.643
Not present	118 (81.4%)	58 (82.9%)	55 (80.0%)		43 (79.6%)	41 (75.9%)	
Any	27 (18.6%)	12 (17.1%)	15 (20.0%)		11 (20.4%)	13 (24.1%)	
Liver metastases				0.884			0.767
Not present	129 (89.9%)	62 (88.6%)	67 (89.3%)		48 (88.9%)	47 (87.0%)	
Any	16 (11.0%)	8 (11.4%)	8 (10.7%)		6 (11.1%)	7 (13.0%)	
PD-L1 TPS				0.221			0.051
<1%	60 (41.4%)	28 (40.0%)	32 (42.7%)		24 (44.4%)	21 (38.9%)	
≥1% and ≤49%	71 (49.0%)	38 (54.3%)	33 (44.0%)		29 (53.7%)	25 (46.3%)	
≥50%	14 (9.7%)	4 (5.7%)	10 (13.3%)		1 (1.8%)	8 (14.8%)	
BMI							
Mean (SD), kg/m^2^	25.8 (5.1)	26.6 (5.2)	25.1 (5.0)	0.109	26.2 (4.6)	25.7 (5.1)	0.511
≥25	76 (52.4%)	37 (52.8%)	39 (52.0%)	0.916	27 (50.0%)	27 (50.0%)	0.999
Smoking habit				0.166			0.999
Never smoker	18 (12.5%)	6 (8.6%)	12 (16.2%)		6 (11.1%)	6 (11.1%)	
Ever	126 (87.5%)	64 (91.4%)	62 (83.8%)		48 (88.9%)	48 (88.9%)	
Type of treatment				0.404			0.054
Nivolumab	88 (60.7%)	46 (65.7%)	42 (56.0%)		38 (70.3%)	30 (55.5%)	
Atezolizumab	12 (8.3%)	6 (8.6%)	6 (8.0%)		6 (11.1%)	3 (5.5%)	
Pembrolizumab	45 (31.0%)	18 (25.7%)	27 (36.0%)		10 (18.5%)	21 (38.9%)	
Reasons for APAP intake				0.461			0.624
Cancer-related	74 (51.0%)	32 (45.7%)	42 (56.0%)		25 (46.3%)	30 (55.5%)	
Treatment-related	50 (34.5%)	27 (38.6%)	23 (30.6%)		21 (38.9%)	17 (31.5%)	
Others	21 (14.5%)	11 (15.7%)	10 (12.3%)		8 (14.8%)	7 (13.0%)	
Corticosteroids (yes)	43 (29.7%)	11 (15.7%)	32 (42.7%)	<0.001	14 (25.9%)	18 (33.3%)	0.399
Systemic antibiotics (yes)	21 (14.5%)	10 (14.3%)	11 (14.7%)	0.948	9 (16.6%)	9 (16.6%)	0.999
PPI (yes)	66 (45.5%	32 (45.7%)	34 (45.3%)	0.963	25 (46.3%)	27 (50.0%)	0.700
Statins (yes)	25 (17.2%)	14 (20.0%)	11 (14.7%)	0.396	9 (16.6%)	6 (11.1%)	0.404
Fibrates (yes)	12 (8.3%)	6 (8.6%)	6 (8%)	0.901	4 (7.4%)	3 (5.5%)	0.696
NSAIDs or ASA (yes)	51 (35.2%)	24 (34.3%)	27 (36.0%)	0.829	20 (37.0%)	20 (37.0%)	0.999
Beta-blockers (yes)	18 (12.4%)	11 (15.7%)	7 (9.3%)	0.244	8 (14.8%)	5 (9.2%)	0.375
ACEi/ARBs (yes)	42 (29.0%)	22 (31.4%)	20 (26.7%)	0.528	18 (33.3%)	14 (25.9%)	0.399
Metformin (yes)	12 (8.3%)	7 (10.%)	5 (6.7%)	0.467	5 (9.2%)	4 (7.4%)	0.728
Oral or transdermal opioids (yes)	52 (35.9%)	32 (45.7%)	20 (26.7%)	0.017	20 (37.0%)	19 (35.1%)	0.841

LAE, low-level acetaminophen (APAP) exposure cohort; HAE, high-level APAP exposure cohort; PSM, propensity score matching; SD, standard deviation; ECOG PS, Eastern Cooperative Oncology Group Performance Status; CNS, central nervous system; PD-L1 TPS, programmed cell death ligand-1 tumor proportion score; BMI, body mass index; PPI, proton pump inhibitors; NSAIDs, nonsteroidal anti-inflammatory drugs; ASA, acetylsalicylic acid; ACEi, angiotensin-converting enzyme inhibitors; and ARBs, angiotensin II type 2 receptor blockers.

**Table 3:** Analysis of correlation between clinical–pathological variables and clinical benefit outcome.

Covariate	First-Line PSM Population					Second-Line PSM Population				
	Univariate Analysis			Multivariate Analysis		Univariate Analysis			Multivariate Analysis	
	NCB N = 26 (100%)	DCB N = 26 (100%)	*p* Value	OR (95% CI)	*p* Value	NCB N = 62 (100%)	DCB N = 46 (100%)	*p* Value	OR (95% CI)	*p* Value
Age			0.999	-	-			0.999	-	
≤70 years	14 (53.8)	13 (50.0%)				28 (45.2%)	20 (43.5%)			
>70 years	12 (45.2%)	13 (50.0%)				34 (54.8%)	26 (56.5%)			
Sex			0.999	-	-			0.828	-	
Female	7 (26.9%)	6 (23.1%)				16 (25.8%)	12 (26.1%)			
Male	19 (73.1%)	20 (76.9%)				46 (74.2%)	34 (73.9%)			
ECOG PS			0.668	-	-			0.617	-	
0 or 1	22 (84.6%)	24 (92.3%)				49 (79.1%)	39 (84.8%)			
2	4 (15.4%)	2 (7.7%)				13 (20.9%)	7 (15.2%)			
Histology			0.703	-	-			0.838	-	
Squamous	3 (11.5%)	5 (19.2%)				20 (32.3%)	16 (34.8%)			
Nonsquamous	23 (88.5%)	21 (80.8%)				42 (67.7%)	30 (65.2%)			
No. of metastatic sites			0.572	-	-			0.018		0.209
≤2	14 (53.8%)	17 (65.4%)				28 (45.2%)	32 (69.6%)		1.00	
>2	12 (46.2%)	9 (34.6%)				34 (54.8%)	14 (30.4%)		0.52 (0.18–1.44)	
Bone metastases			0.703	-	-			0.032		0.171
Not present	21 (80.3%)	23 (88.5%)				44 (71%)	41 (89.1%)		1.00	
Any	5 (19.2%)	3 (11.5%)				18 (29.0%)	5 (10.9%)		0.38 (0.10–1.50)	
SNC metastases			0.523	-	-			0.354	-	-
Not present	18 (69.2%)	21 (80.8%)				46 (74.2%)	38 (82.6%)			
Any	8 (30.8%)	5 (19.2%)				16 (25.8%)	8 (17.4%)			
Liver metastases			0.703	-	-			0.999	-	-
Not present	2 (80.%)	23 (80.8%)				54 (87.1%)	41 (89.1%)			
Any	5 (19.2%)	3 (11.5%)				8 (12.9%)	5 (10.9%)			
PD-L1 TPS			0.210	-	-			0.119	-	-
<1%	7 (26.9%)	5 (19.2%)				31 (50.0%)	14 (30.4%)			
≥1% and ≤49%	1 (3.8%)	5 (19.2%)				27 (43.5%)	27 (58.7%)			
≥50%	18 (69.2%)	16 (61.5%)				4 (6.5%)	5 (10.9%)			
BMI			0.781	-	-			0.560	-	-
≥25 kg/m^2^	12 (46.2%)	13 (50.0%)				29 (47.5%)	25 (53.2%)			
Smoking habit			0.999	-	-			0.758	-	-
Never smoker	2 (7.7%)	3 (11.5%)				8 (12.9%)	4 (8.7%)			
Ever	24 (92.3%)	23 (88.5%)				54 (87.1%)	42 (91.3%)			
Type of treatment			0.313	-	-	-	-	-	-	-
Pembrolizumab	19 (73.1%)	16 (61.5%)								
Pemetrexed-based	7 (26.9%)	8 (30.8%)								
Paclitaxel-based	-	2 (7.7%)								
Type of treatment	-	-	-	-	-			0.991	-	-
Nivolumab						39 (62.9%)	29 (63.0%)			
Atezolizumab						5 (8.1%)	4 (8.7%)			
Pembrolizumab						18 (29.0%)	13 (28.3%)			
APAP exposure			0.012		0.008			<0.001		<0.001
Low	8 (30.8%)	18 (69.2%)		1.00		20 (32.2%)	34 (73.9%)		1.00	
High	18 (69.2%)	8 (30.8%)		0.18 (0.05–0.64)		42 (67.8%)	12 (26.1%)		0.17 (0.07–0.43)	
Reasons for APAP intake			0.165	-	-			0.182	-	-
Cancer-related	15 (57.7%)	10 (38.5%)				35 (56.4%)	20 (43.5%)			
Cancer-unrelated	11 (42.3%)	16 (61.5%)				27 (43.6%)	26 (56.5%)			
Corticosteroids			0.040		0.027			0.019		0.033
No	13 (50.0%)	21 (80.8%)		1.00		38 (61.3%)	38 (82.6%)		1.00	
Yes	13 (50.0%)	5 (19.2%)		0.21 (0.05–0.84)		24 (39.3%)	8 (17.4%)		0.33 (0.12–0.91)	
Systemic antibiotics (yes)	6 (23.1%)	3 (11.5%)	0.465	-	-	9 (14.8%)	9 (19.1%)	0.608	-	-
PPI (yes)	13 (50.0%)	11 (42.3%)	0.781	-	-	27 (44.3%)	25 (53.2%)	0.438	-	-
Statins (yes)	7 (26.9%)	7 (26.9%)	0.999	-	-	9 (14.8%)	6 (12.8%)	0.999	-	-
Fibrates (yes)	3 (11.5%)	3 (11.5%)	0.999	-	-	3 (4.9%)	4 (8.5%)	0.466	-	-
NSAIDs or ASA (yes)	10 (38.5%)	10 (38.5%)	0.999	-	-	19 (31.1%)	21 (44.7%)	0.165	-	-
Beta-blockers (yes)	12 (46.2%)	6 (23.1%)	0.144	-	-	8 (13.1%)	5 (10.6%)	0.773	-	-
ACEi/ARBs (yes)	11 (42.3%)	10 (38.5%)	0.999	-	-	16 (26.2%)	16 (34.0%)	0.402	-	-
Metformin (yes)	4 (15.4%)	2 (7.7%)	0.668	-	-	5 (8.2%)	4 (8.5%)	0.999	-	-
Oral or transdermal opioids (yes)	12 (46.2%)	10 (38.5%)	0.779	-	-	23 (37.7%)	16 (34%)	0.840	-	-

PSM, propensity score matching; NCB, No Control Benefit; DCB, Durable Control Benefit; OR, odds ratio; CI, confidence interval; ECOG PS, Eastern Cooperative Oncology Group Performance Status; CNS, central nervous system; PD-L1 TPS, programmed cell death ligand 1 tumor proportion score; BMI, body mass index; LAE, low-level acetaminophen (APAP) exposure cohort; HAE, high-level APAP exposure cohort; PPI, proton pump inhibitors; NSAIDs, nonsteroidal anti-inflammatory drugs; ASA, acetylsalicylic acid; ACEi, angiotensin-converting enzyme inhibitors; and ARBs, angiotensin II type 2 receptor blockers.

**Table 4:** Analysis of survival in a first-line treatment setting.

Covariate	Progression-Free Survival				Overall Survival			
	Univariate Analysis		Multivariate Analysis		Univariate Analysis		Multivariate Analysis	
	Median (95% CI), Months	*p* Value	HR (95% CI)	*p* Value	Median (95% CI), Months	*p* Value	HR (95% CI)	*p* Value
APAP exposure		0.002		0.001		0.002		0.003
Low	16.5 (9.5–23.4)		1.00		20.5 (14.2–26.7)		1.00	
High	5.2 (3.3–7.0)		0.34 (0.18–0.66)		9.3 (7.8–10.8)		0.36 (0.18–0.71)	
Corticosteroids		0.002		0.002		0.003		0.002
No	11.5 (7.3–15.7)		1.00		17.9 (15.4–20.4)		1.00	
Yes	5.7 (3.8–7.5)		0.33 (0.17–0.66)		9.8 (9.3–10.3)		0.33 (0.16–0.67)	

CI, confidence interval; HR, hazard ratio; and APAP, acetaminophen.

**Table 5:** Analysis of survival in a second-line treatment setting.

Covariate	Progression-Free Survival				Overall Survival			
	Univariate Analysis		Multivariate Analysis		Univariate Analysis		Multivariate Analysis	
	Median (95% CI), Months	*p* Value	HR (95% CI)	*p* Value	Median (95% CI), Months	*p* Value	HR (95% CI)	*p* Value
APAP exposure		0.016		0.016		0.005		0.005
Low	7.8 (1.4–14.2)		1.00		12.8 (6.6–19.0)		1.00	
High	3.3 (2.3–4.3)		0.61 (0.40–0.91)		6.5 (3.6–9.4)		0.54 (0.35–0.83)	
Corticosteroids		0.003		0.002		0.045		0.012
No	6.3 (3.5–9.0)		1.00		10.8 (6.8–14.8)		1.00	
Yes	2.9 (1.6–4.2)		0.50 (0.32–0.78)		4.4 (2.7–6.1)		0.56 (0.35–0.88)	

CI, confidence interval; HR, hazard ratio; and APAP, acetaminophen.

## Data Availability

The datasets generated and analyzed during the current study are available from the corresponding author upon reasonable request.

## Supplementary Materials

The following supporting information can be downloaded at: https://www.mdpi.com/article/10.3390/curroncol30090589/s1, Figure S1: Progression-free survival depending on clinical benefit outcome; Figure S2: Overall survival depending on clinical benefit outcome.
