# Supplementary material for: Effects of Acetaminophen Exposure on Outcomes of Patients Receiving Immune Checkpoint Inhibitors for Advanced Non-Small-Cell Lung Cancer: A Propensity Score-Matched Analysis
Source: Curr Oncol. 2023 Sep 1;30(9):8117–33. doi: 10.3390/curroncol30090589 (PMC10527930; doi:10.3390/curroncol30090589)
Supplement: Supplementary file 1 [file curroncol-30-00589-s001.zip › curroncol-2547117-supplementary.pdf]

SUPPLEMENTARY MATERIAL

Effects of acetaminophen exposure on outcomes of patients receiving immune checkpoint inhibitors for advanced non-small-cell lung cancer: a propensity score-matched analysis.

Fabrizio Nelli[1], Antonella Virtuoso[1], Diana Giannarelli[2], Agnese Fabbri[3], Julio Rodrigo Giron Berrios[3], Eleonora Marrucci[3], Cristina Fiore[3], and Enzo Maria Ruggeri[3]

[1] Department of Oncology and Hematology, Thoracic Oncology Unit, Central Hospital of Belcolle, Viterbo, Italy

[2] Biostatistics Unit, Scientific Directorate, Fondazione Policlinico Universitario A. Gemelli, IRCCS, Rome, Italy

[3] Department of Oncology and Hematology, Medical Oncology Unit, Central Hospital of Belcolle, Viterbo, Italy

*** Correspondence:** Fabrizio Nelli, MD; Department of Oncology and Hematology, Thoracic Oncology Unit, Central Hospital of Belcolle, Strada Sammartinese snc, 01100 Viterbo, Italy, Phone +390761339055, Fax +390761339039, e-mail: fabrizio.nelli@asl.vt.it, ORCID iD: 0000-0001-8374-1362

Supplementary Figure 1. Progression-free survival depending on clinical benefit outcome

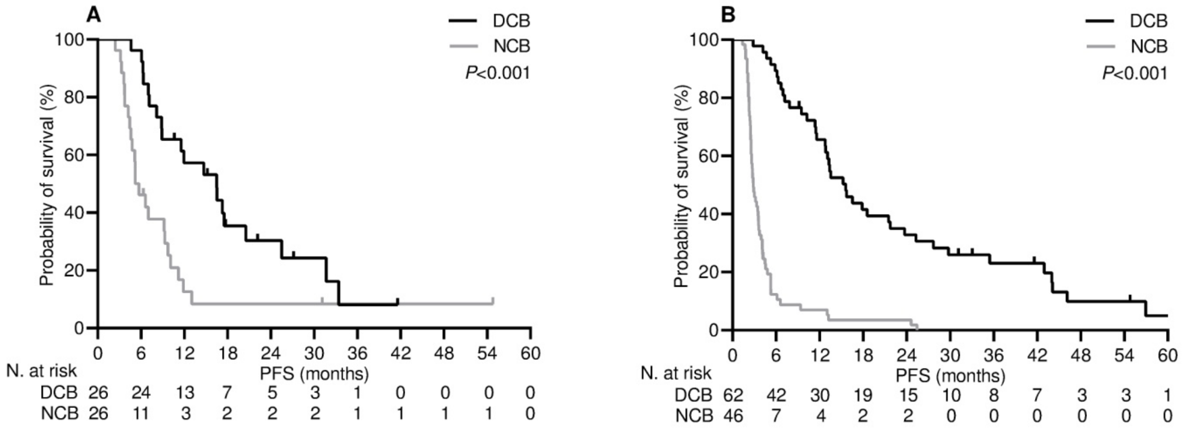

(A) First-line treatment setting and (B) second-line treatment setting: DCB (durable clinical benefit) vs. NCB (no clinical benefit).

Supplementary Figure 2. Overall survival depending on clinical benefit outcome

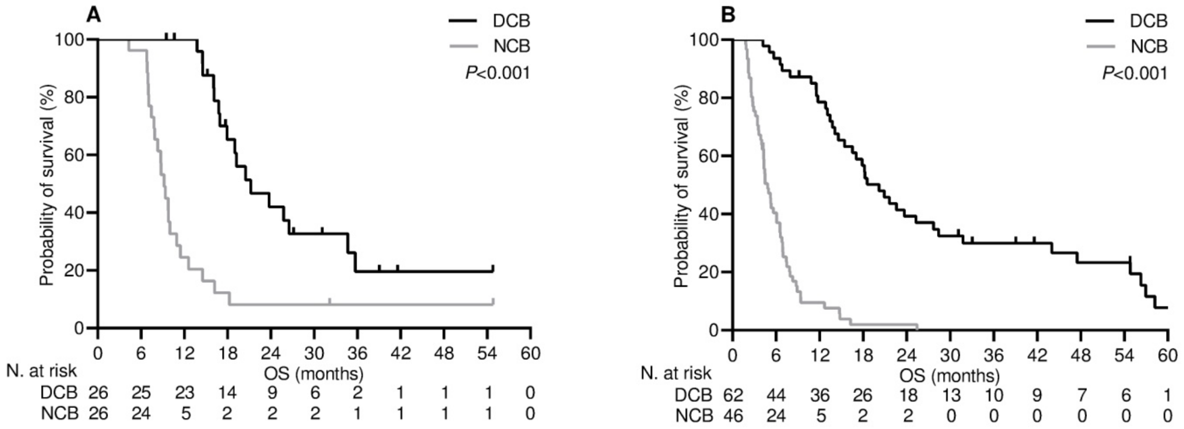

(A) First-line treatment setting and (B) second-line treatment setting: DCB (durable clinical benefit) vs. NCB (no clinical benefit).
